# Landmarks or panoramas: what do navigating ants attend to for guidance?

**DOI:** 10.1186/1742-9994-8-21

**Published:** 2011-08-27

**Authors:** Antoine Wystrach, Guy Beugnon, Ken Cheng

**Affiliations:** 1 Department of Biological Sciences, Macquarie University, Sydney, NSW 2109 Australia; 2 Centre de Recherches sur la Cognition Animale, CNRS, UMR 5169, Université Paul-Sabatier, Toulouse F-31062, France

**Keywords:** ant, insect navigation, panoramic views, landmark, route learning

## Abstract

**Background:**

Insects are known to rely on terrestrial landmarks for navigation. Landmarks are used to chart a route or pinpoint a goal. The distant panorama, however, is often thought not to guide navigation directly during a familiar journey, but to act as a contextual cue that primes the correct memory of the landmarks.

**Results:**

We provided *Melophorus bagoti *ants with a huge artificial landmark located right near the nest entrance to find out whether navigating ants focus on such a prominent visual landmark for homing guidance. When the landmark was displaced by small or large distances, ant routes were affected differently. Certain behaviours appeared inconsistent with the hypothesis that guidance was based on the landmark only. Instead, comparisons of panoramic images recorded on the field, encompassing both landmark and distal panorama, could explain most aspects of the ant behaviours.

**Conclusion:**

Ants navigating along a familiar route do not focus on obvious landmarks or filter out distal panoramic cues, but appear to be guided by cues covering a large area of their panoramic visual field, including both landmarks and distal panorama. Using panoramic views seems an appropriate strategy to cope with the complexity of natural scenes and the poor resolution of insects' eyes. The ability to isolate landmarks from the rest of a scene may be beyond the capacity of animals that do not possess a dedicated object-perception visual stream like primates.

## Introduction

Many insects use terrestrial objects -- landmarks -- for navigation. Ants and bees in particular are known to rely on landmarks both to pinpoint a goal [1-4], and also to chart routes, which are typically idiosyncratic paths through a landscape dotted with landmarks [5-9]. Knowledge about how insects exploit such landmark information comes mostly from studies conducted in visually controlled and impoverished conditions, like experimental rooms or deserts, where the salience of many potential cues is minimal and only experimental landmarks are made prominent [1,2,4,10-14].

Concurrently, studies conducted in visually rich environments suggested that ants and bees ignore the features of familiar landmarks if they are presented within a wrong panoramic context [15]. This led to the idea that panoramas and landmarks are different cues that have different functions: a class of theories claims that the panorama serves as a contextual cue that triggers the recall of the appropriate landmark memory, on which guidance is based [16-18]. The segregation between landmark and panorama seems striking in these experimental conditions. This class of theories, however, faces the question of how insects segregate contextual cues and landmarks in natural environments, with complex depth structures. One theoretical proposal is that the amount of motion parallax is used as a depth cue to filter out distant landmarks [19]. Insects are known to use motion parallax as a depth cue [20-25]. But whether insects use such depth information to segregate out landmarks has not been determined empirically.

It has also been suggested that insects may not segregate landmarks from the panorama at all but are guided instead by cues widespread on their panoramic visual field, which encompass both landmarks and panorama [26-32]. Some results support this class of theories. For instance, multiple landmarks [1,2,33] but also landmarks and panorama [9,34] seem to be "bound together" in insect memories. An imitation of the skyline (elevations of surrounding terrestrial objects) is sufficient for orientation in one species of desert ants [26]. In some cases [14,30], the ants proved able to navigate robustly using pretty much plain white walls or curtains. Such performance can be readily explained by the use of panoramic views that encompass the global shape of the arena [35]. Yet it is difficult to create experimental conditions in which the two classes of theories make different predictions. For example, in a previous study, many manipulations on ants' home routes were conducted [9]. But both classes of theories could account equally well for the large body of results.

**Figure 1:**
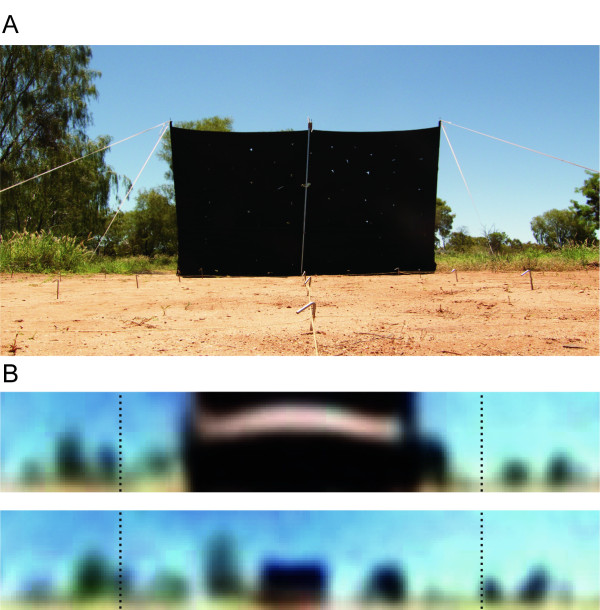
**Photos of the experimental set up with the landmark in training position**. A. Picture taken 5 m from the nest. B. Ant's-eye picture (300°, resolution of 4° [49]) taken 5 m from the nest (bottom) or at the nest position (top). The dashed lines delimit 180°. The landmark was located 90 cm behind the nest while the closest tree (on the left of the picture A) was located 14 m away from the nest. The panorama was thus providing very little dynamic change compared to the landmark for ants approaching their nest.

We here investigate the effect of displacing a prominent landmark within natural surroundings. A huge black landmark was placed immediately behind a nest entrance of *Melophorus bagoti *ants (Figure 1). Standing in a flat area devoid of proximal trees, the landmark was designed to stick out from the rest of the panorama, and thus to be as easy as possible for an insect to learn, memorise, and extract from the rest of the scenery. We analysed the paths displayed by the ants in response to displacements of the landmark. In parallel, we recorded panoramic 'ant's eye' images in order to quantify the panoramic alteration of the scenery caused by the landmark displacements. This approach allowed us to relate the ants' behaviour not only to the landmark, but to the whole panoramic scene, providing us with insight on whether navigating ants were focusing on the landmark or using cues widespread on their panoramic visual field.

## Results

### Panoramic pictures: image difference distribution

**Figure 2:**
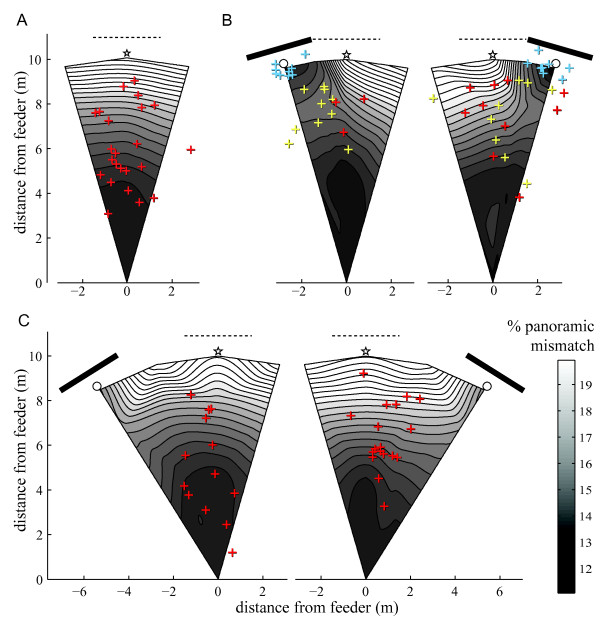
**Maps of panoramic mismatches and first U-turn locations**. The maps result from comparing panoramic pictures taken during Removal of the landmark (A), Rotation 16° (B) and Rotation 32° (C) tests with reference pictures from the training condition. '% of panoramic mismatch' indicates the percentage of mismatching pixel across the image. Locations for comparisons are shown in the Additional file 1. Mismatch levels were then interpolated between those locations (triangle-based cubic interpolation). The darker the shade, the lower the mismatch between views. Each cross represents the location of the first U-turn (walking at least 20 cm back towards the feeder within 50 cm of displacement) of the ants. Red crosses: first U-turns of ants that never searched densely in front of the landmark. Yellow crosses: first U-turn of ants that displayed a U-turn before searching in front of the landmark. Blue crosses: first U-turn of ants that displayed no U-turn before searching in front of the landmark (Blue crosses thus correspond to the beginning of the search). Bar: landmark position during test. Dashed line: landmark position during training. Stars: nest position. White circle: fictive nest position relative to the landmark.

We quantified the alteration of the visual panorama created by the different displacements of the landmark (Figure 2). Across multiple positions, we recorded and compared panoramic images taken with the landmark either in the training position (reference scenery) or displaced (test scenery) (see Additional file 1). The panoramic image difference between reference and test scenery across the field can then be calculated [32]. At first, the landmark was shifted into a distant area (roughly 100 m away). The picture comparisons revealed high image differences between the training and distant test field. Indeed, even in front of the landmark, a great part of the panoramic view is very different from that found at an equivalent position on the training field. Within the training area, removing the landmark does not significantly alter the view at the beginning of the route but results in high image differences near the nest position (Figure 2A). Indeed, the visual area covered by the landmark (or here, absence of landmark) is negligible at the feeder but increases as a tangent function as one approaches the nest (Figure 1, see also Additional file 1). Similarly, shifting the landmark by 16° or 32° does not significantly alter the view at the beginning of the route but creates high image differences at the real nest position.

The 16° displacement creates a region of high image difference in the area opposite to the displaced landmark. This results in a valley of lower image differences between the feeder and the landmark. Within this valley, a zone of higher mismatch is located around 7-8 m on the way towards the 16° displaced landmark (Figure 2B). The presence of this latter zone of mismatch is easily explained. As one moves from the feeder towards the displaced landmark, the image differences result from two competing factors: the landmark and the rest of the panorama. As a result of moving away from the training direction, the perceived distant panorama (i.e., all the scenery except the landmark) becomes more and more altered, thus steadily increasing the mismatch. The landmark, however, matches its target counterpart perfectly, and although very small at the beginning of the route (filling < 5% of the azimuth), it increases in size sharply with distance, thereby minimizing the global mismatch. In combining landmark and panorama, the panoramic image differences grows as an ant travels from the feeder towards the landmark until a point of maximum mismatch (around 7-8 m along the feeder nest axis), beyond which the increasing size of the matching landmark diminishes the global mismatch (Figure 2B).

It is important to emphasise that the distributions of image differences presented here are not intended to model a particular homing strategy such as matching gradient descent, but simply allowed us to quantify the modification of the panoramic scenery the ants were subjected to during the tests. Whatever the actual process involved, any guidance strategy based on panoramic input should lead to disrupted behaviour if the global scenery is too much altered. Therefore, if ants are guided by panoramic views, they should not be able to reach the nest position in any of the tests conditions, as all of them present substantial panoramic image differences around the nest location. With 16° displacements of the landmark, however, the ants may end up searching in front of the displaced landmark, but their approaching route should then be altered while crossing the hill of high mismatch located at around 7-8 m.

### Panoramic pictures: rotational image difference

**Figure 3:**
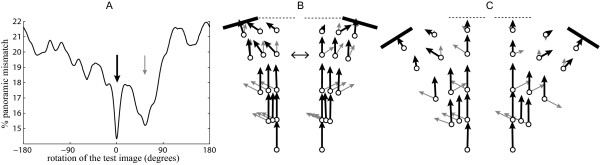
**Rotational image differences**. Images from the test conditions (i.e., current view) are compared with the 'references images' from the training condition (i.e., memorised view) for every possible rotation. The 'reference images' have been taken along the feeder-nest line and are all facing towards the nest. A. Example of the image differences distribution between a test image and the 'reference image' as a function of the rotation of the test image. The two lowest choices of image differences are indicated by the black (best choice) and grey (second best choice) arrows. '% of panoramic mismatch' indicates the percentage of mismatching pixel across the image. B, C. Black and grey arrows of a given location represent respectively the best and second best matching rotation of a given location when the landmark is displaced by 16° (B) and 32° (C). The length of the arrows is proportional to the matching value.

Panoramic images can be rotated until they produce the best matching to the reference image. Here, rotational IDFs (i.e., Image Difference as a Function of the rotation) presented often two distinct best choices for matching (Figure 3A). One choice (distant panorama choice) was generally obtained while facing the same direction as during training, because the distant panoramas of both images overlap well. The other choice (landmark choice) was generally obtained while facing roughly towards the displaced landmark, because the landmarks of both images are superimposed.

**Figure 4:**
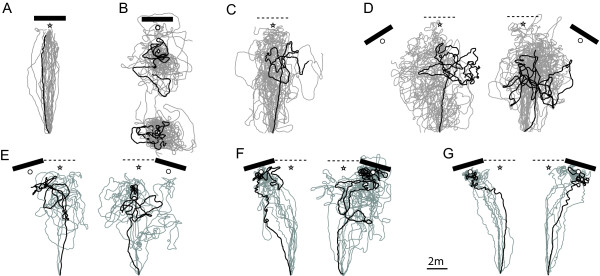
**Test paths of individual ants**. Ant were captured at the nest and released at the feeder (10 m away from the nest), with (A) the landmark in the same position as during training; (B) the landmark placed in a distant area (ant released either 2 m or 10 m in front of the fictive nest entrance); (C) the landmark removed; (D) the landmark rotated 32° away from the feeder-nest line (centred on the feeder) to the left or the right; (E, F, G) the landmark rotated 16° for 3 categories of ants: (E) ants that never display a dense 'nest-search' in front of the landmark; (F) ants that displayed a nest-search in front of the landmark but showed U-turns during their approach; (G) ants that displayed a nest-search in front of the landmark but showed no U-turn during their approach. Each path represents an individual ant, with one of them chosen at random highlighted in black. Bar: landmark position on the test (3 m wide). Dashed line: landmark position during training. Star: nest position. White circle: fictive nest position relative to the landmark. Diamond: release point on the distant test field.

At the beginning of the route, the 'distant panorama choice' provides a better matching value than the 'landmark choice' (Figure 3B, C) because the landmark appears very small and the distant panorama covers most of the view. However, as one travels towards the nest, the apparent size of the landmark increases and the part of the field of view covered by the distant panorama decreases. Therefore, the 'landmark choice' matching quality grows and the 'distant panorama choice' decreases in importance. Ants travelling towards their nest in the training direction may thus suddenly switch in orientation when the image difference along the 'distant panorama choice' becomes too bad or when the 'landmark choice' direction becomes better. Such a switch towards the landmark direction does not imply that the ant is now attending to the landmark, but just that the panoramic image difference is lower while facing in that new direction. Ants may also 'hesitate' between the two directions when they provide equivalent matching quality, leading potentially to wiggling paths for that part of the route (see examples Figure 4G).

Interestingly, a side difference arises in the 16° conditions. When the landmark is displaced to the left, facing the landmark becomes the best matching rotation at earlier locations than when the landmark is displaced to the right (Figure 3B). Some ants might thus continue to walk in the training direction longer when the landmark is displaced to the right.

Following purely the strategy of walking in the best matching direction should nonetheless lead the ant to the displaced landmark in the 16° condition (Figure 3B) and to the nest or the landmark in the 32° condition (Figure 3C). However, the actual image difference value of the selected direction might be important too. Ants might stop following the best matching direction if the image difference is considered too high (Figure 2 displays the distribution of the actual best matching values).

### Ant responses: control condition

Ants homing from the feeder were captured just before reaching their nest in front of the landmark, and released again at the feeder location. When the landmark was left at its original position, the ants ran their route home again readily (Figure 4A) showing that they were guided by the perceived scenery and were not affected by potentially conflicting information provided by their path integrator. However, changing either the presence or the position of the landmark affected homing performance adversely, showing that the ants were affected by such alteration of the scenery.

### Ant responses: distant area, 32° displacements and removal of the landmark

When the landmark was translated to a distant area presenting an unfamiliar panorama, the ants released at 10 m (i.e., fictive feeder) or 2 m in front of the fictive nest engaged immediately in a search pattern at the release point and none of them (0 out of 16 and 0 out of 15 respectively) went searching at the fictive nest relative to the landmark (Figure 4B).

On the training field, with the landmark removed (Figure 4C) or displaced by 32° (Figure 4D), the ants tended to run a first relatively straight segment but then displayed a U-turn on average half way from the nest and started searching. None of these ants (No-landmark: 0 out of 23; Left and Right 32° displacements: 0 out of 31) found the nest or reached the fictive nest in front of the displaced landmark (within 3 min). Interestingly, the approaches were on average centred along the feeder-nest axis in the no-landmark condition, but were a little bit skewed towards the displaced landmark in the 32° condition (Additional file 2). The searches appeared centred on the first U-turn, but, interestingly, showed a larger spread than the searches displayed on the distant area (Additional file 2).

### Ant responses: 16° displacements of the landmark

With a smaller displacement of the landmark (16°), the ants displayed different behaviours, which we categorised into 3 groups (Figure 4E, F, G). Some ants (13 out 49) never reached the nest or searched for it in front of the displaced landmark (Figure 4E). The others (36 out of 49 individuals) eventually aimed at the landmark and displayed a dense search for the nest in front of it (Figure 4F, G). Determining whether (Figure 4F, G) or not (Figure 4E) an ant searched at the goal proved completely unambiguous, as two independent judges could agree completely: the 'nest-search' pattern would suddenly get much denser and the ants would not leave the area in front of the landmark for several minutes.

The 36 ants that searched for the nest (i.e., dense nest-search) in front of the landmark were categorised in two groups depending on whether or not they displayed U-turns while approaching the landmark. U-turns consisted of more than a sharp turn, but also the stipulation that the ant walk back in a direction at least 113 degrees away from the training direction (i.e., went at least 20 cm down along the Y axis within 50 cm of travel). As a result, an ant could display very sharp turns to the left and to the right without being considered as displaying U-turns.

Around half of these ants (19 out of 36) displayed at least one U-turn before reaching the displaced landmark (Figure 4F), a much higher proportion than in the control group returning under training conditions (Fisher's exact test: 19/36 vs. 1/25, odds ratio 25.56, p < 0.0001). Interestingly, their first U-turns were not located randomly along the route (Chi-square against random distribution across categories of 1 m: χ^2 ^= 30, df = 9, p < 0.0001) but occurred mostly between 7 m and 8 m away from the feeder (first U-turn average distance from the feeder ± sd: 7.43 ± 1.48 m) (Figure 2B yellow crosses, Figure 4F).

**Figure 5:**
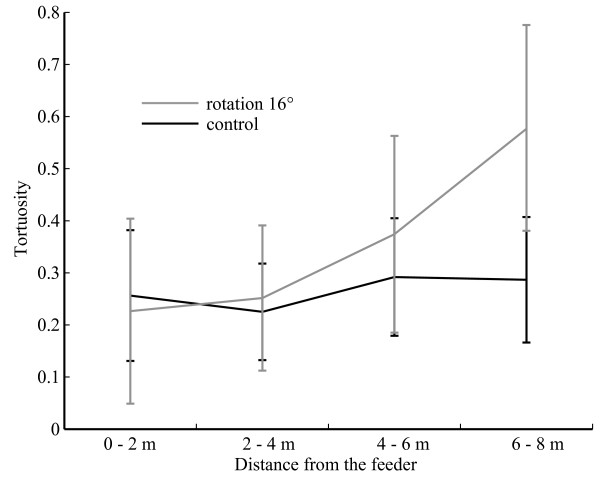
**Tortuosity along the path**. Index of path tortuosity at different distances away from the feeder (M ± sem) for ants from the control group (in black) and from the Rotation 16° condition (in grey) that displayed no U-turn before searching in front of the landmark. The tortuosity index corresponds to the averaged absolute angle (in radians) between the directions of successive chunks of 20-cm line segments connecting points on the path. A circle of 20 cm radius was placed at the starting point of the digitised path, and where the circle intersected the path defined the first segment. The circle was then placed at the end of segment 1 to define segment 2, etc.

The other half (17 out of 36) approached the landmark without displaying any U-turns. The first U-turn of these 17 individuals occurred right in front of the landmark (Figure 2B blue crosses, Figure 4G) and, rather than showing uncertainty en route, corresponds to the beginning of the characteristic dense search for the nest entrance. However, a closer look at the approach of those individuals revealed an increasing tortuosity that reaches its maximum around 7 to 8 m away from the feeder, a pattern that was not observed in the control group (ANOVA groups*distances: n = 17+25, F = 9.008, p = 0.0001; between groups: F = 12.971, p = 0.0009) (Figure 5).

Overall, even though most ants searched for the nest in front of the 16°-displaced landmark, its displacement notably affected their approach. Their paths were more tortuous than in the control condition: wiggles and U-turns were strongest around 7 to 8 m away from the feeder.

### Path tortuosity and compass direction

To test whether or not this degradation was due to the fact that the ants in the 16° condition were led in a slightly different compass direction than during training, we focused on individuals that displayed long segments oriented towards the displaced landmark. Some ant paths (17 out of 48) presented a neat transition in the direction of travel, with a first segment oriented towards the nest and a second segment oriented towards the landmark (see Additional file 3 for examples). The switch in direction occurred on average around 5 m away from the feeder (average distance from the feeder ± sd: 5.1 ± 1.3 m). Around half of those ants (8 out of 17) displayed a first U-turn while approaching the landmark. Those first U-turns did not occur immediately after the switch towards the landmark as it would be expected if the path disruption was due to the new compass direction of travel, but several meters thereafter (average distance between switch and first Uturn ± sd: 3.8 ± 0.9 m), that is, around 7-8 m away from the feeder (average distance of the first U-turns from the feeder ± sd: 7.9 ± 1.1 m).

Other ants (8 out of 48) headed towards the 16°-displaced landmark from the start (see Additional file 3 for examples). Although the direction of travel was similarly oriented towards the landmark all along their approach (heading direction: paired sample t test: 0-4 meter vs. 4-8 meter, t = -0.508, p = 0.627), the tortuosity of their paths increased significantly in the second half of the journey (tortuosity: paired sample t test: 0-4 meter vs. 4-8 meter, t = -4.635, p = 0.002) and half of them (4 out of 8) also displayed a first U-turn after 5 m of travel towards the landmark (first U-turn average distance from the feeder ± sd: 6.5 ± 1.3).

Overall, ants were not equally perturbed everywhere along their way towards the landmark. Their paths were disrupted mostly around 7 m away from the feeder, independently of the ant's compass direction of travel. It seems therefore unlikely that the observed degradation of the path results from a discrepancy between the landmark direction and a memorised celestial compass information.

### Side differences

In both 16° and 32° conditions, displacing the landmark to the left or to the right had different effects on the ants' first U-turn location. In 16° conditions, U-turn location differences appeared along the x-axis. When the landmark was displaced to the right, U-turns occurred closer to the feeder-nest axis and further away from the landmark side than when the landmark was displaced to the left (t-test independent samples (values mirrored for one side): t = 4.254, p = 0.0002). Remarkably, such a difference was predicted by the panoramic image comparisons (see "rotational matching of panoramic views"). Along the y-axis, the distribution of first U-turns were similar on average (t-test independent samples: t = 1.587, p = 0.1234) but were more spread in the 16°Right condition (Levene's test: F = 6.376, p = 0.0170).

With 32° displacement of the landmark, no side differences in U-turns distribution appeared along the x-axis (t-test independent samples: t = -1.208, p = 0.2368). Along the y-axis, however, U-turns occurred significantly earlier (t-test independent samples: t = 14.047, p < 0.0001) and were significantly more scattered (Levene's test: F = 6.128, p = 0.0190) when the landmark was displaced to the right.

## Discussion

*Melophorus bagoti *lives a habitat full of landmark information such as bush, trees or distant cliffs, and evolution has tuned those ants to learn quickly [36] and rely heavily on the so called 'landmark information' [7]. We here investigate whether navigating ants functionally segregate the perceived scenery into landmarks for guidance and the panorama as contextual cue. Such theories infer that, for the animal, the given landmark is somewhat isolated from the rest of the panorama. For this purpose, we gave ants every incentive to isolate a landmark from the panorama by choosing an area devoid of proximal trees, by clearing that area of any proximal clutter and providing them with a particularly prominent artificial landmark at the nest entrance.

### Initial segments and searches

The ants accustomed to the landmark behind their nest were captured as zero-vector ants (i.e., just before reaching their nest entrance) and released again at the feeder position. Because the 'zero state' of their path integrator cannot provide them with a homing direction, zero-vector ants have to rely on the visual surroundings to home. In a landmark rich habitat, the recognition of the surrounding overrides completely the information given by the path integrator [7,9,37]. It is therefore not surprising that when the landmark was left at its original position, the recognition of familiar surroundings led zero-vector *M.bagoti *ants to run their home route again readily and thoroughly (Figure 4A).

When the landmark was displaced from the training position, however, the ant routes were notably altered, revealing that such modification of the scenery affected their homing. When released on the distant test field, the large landmark was not used: ants engaged immediately in a systematic search around the release point (Figure 4B) as they typically do when released in an unfamiliar environment [38]. When the landmark was removed from the training field or displaced by 16° or 32° to the sides, most of the ants ran first a relatively straight segment, showing that they recognised the scenery at the beginning of the route (Figure 4C, D, E, F, G). Whether the ants recalled a local vector (i.e., segment of travel based on compass information) or used a view based matching strategy to achieve this first segment cannot be properly disentangled here. But previous work on this species showed that panorama can be matched and used independently of the compass direction [26] stressing the use of a view based matching strategy rather than a local vector. Moreover, some approaches were here skewed towards the landmark in both 16° and 32° conditions (see Additional file 2), contesting the hypothesis of a pure local vector.

The ants from the 32° displacements (Figure 4D) or no-landmark conditions (Figure 4C) engaged in winding search loops on average half-way to the nest. None searched at the real nest or at the fictive nest position in front of the landmark. Interestingly, these searches were more spread than the systematic search displayed on unfamiliar terrain, revealing that other factors, possibly view based matching or compass information, were influencing the search pattern (Additional file 2).

### Guidance is not focused on the landmark

The hypothesis assuming that the distant panorama is not used for guidance but as a contextual cue provides an explanation for the behaviours described above. The panoramic context could be seen as delivering a negative verdict, rendering the landmark not worth approaching, and triggering the observed search behaviours. However, two pieces of evidence show that guidance was not purely based on the landmark, and that ants were attending simultaneously to other cues from the panorama.

Firstly, displacing the landmark to the left or to the right had different effects on the ants. A 16° displacement to the left led the ants to meander more towards the landmark and less towards the feeder-nest middle line than a 16° displacement to the right. And a 32° displacement to the left had an earlier impact on the ants' paths than a 32° displacement to the right. Since the landmark presented highly contrasted edges against the background, such side differences should not have arisen if guidance was purely based on the landmark.

Secondly, in the 16° displacement condition, some ants ended up searching for their nest in front of the landmark, but their approach did not resemble the straight approach found in control ants (compare Figure 4A with Figures 4F, G). Instead, they exhibited behaviours indicative of 'uncertainty': they U-turned or showed more tortuous paths during their approach (Figure 4F, G). The presence of this uncertainty in the ant paths was independent of their direction of travel, rejecting the hypothesis that uncertainty was resulting purely from a conflict with a stored local vector (i.e., a memorised compass direction) pointing along the training direction. Consistent with this, previous work showed that *M.bagoti *can readily match and use familiar panorama presented in a wrong compass direction [26]. As ants reached -- and therefore used -- the displaced landmark, the path uncertainty observed cannot be attributed to a negative verdict of a hypothetical contextual cue either. Such path uncertainty must therefore result from an alteration of the terrestrial cues the ants were using for guidance. As the highly contrasted landmark was not altered in itself, we can conclude that guidance was simultaneously based on other terrestrial cues.

### Functional segregation landmarks/panorama or panoramic views?

As guidance was not focused on the landmark only, the class of theories assuming a functional segregation between panorama (as context) and landmarks (as guidance cues) needs to invoke other processes like the simultaneous use of other landmarks extracted from the distant landscape for guidance and not for context. But then the process of deciding which landmarks are to be used for guidance and which ones are used as contextual cues appears complex and cannot be based on a simplistic distinction between proximal landmarks and distal panorama. We find it most parsimonious to account for the results by proposing that the ants in our experiment were using guidance strategies based on large panoramic views, without summoning the need to segregate such panoramic views into context and landmarks.

**Figure 6:**
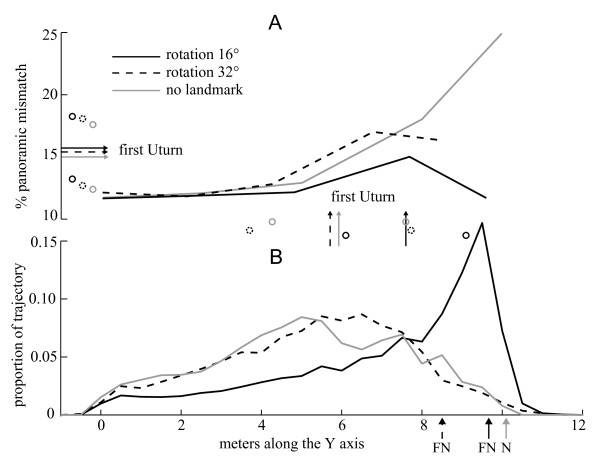
**Panoramic mismatches and search distribution along the Y-axis**. A. Panoramic mismatch along the feeder-landmark line for the Rotation 16° and 32° tests and along the feeder-nest line for the no-landmark test. The vertical arrows represent the average position of the first U-turn displayed by each ant (counting only ants that displayed U-turns before reaching the landmark) (ANOVA between groups: F = 11.096; p < 0.0001; all pairs Tukey's post hoc: Rotation16° a, Rotation32° b, no-landmark b). The horizontal arrows represent the average value of mismatch where the first U-turns were displayed (inferred from the 2D distribution of image difference, Figure 2) (ANOVA between groups: F = 0.3823; p = 0.6835). The open circles on the side of each arrow indicate the inter-individual standard deviation. B. Search distribution of the ants along the Y-axis. Paths were limited to the first 30 m travelled. The relative distribution was first calculated for each individual and then pooled for each condition so that each individual ant contributed equally to the distribution. The arrows indicate the position of the nest (for the no-landmark condition) or the fictive nest in front of the landmark (for Rotation 16° and 32° conditions).

By comparing panoramic images in simple ways, we could explain here the sharp transitions in the direction of travel observed, the presence of wiggling paths at particular locations, some of the differences observed when displacing the landmark to the left or to right (see 'Rotational matching of panoramic views' in Results), as well as why the ant routes were disrupted at different locations across groups (U-turns and searching) (see 'Panoramic image difference distribution' in Results). The correspondence between the regions where the ants' travel was disrupted and the regions of high panoramic image differences (on average ~15% in this case, but with individual variation) (Figure 6) suggests that guidance cues must be widespread on the ants' panoramic visual field.

### How to match panoramic views?

Despite a great amount of work [31,32,35,39-42] how insects match memorised and current views to produce such efficient navigational behaviours is far from fully understood. Recent evidence shows that ants are able to align their body in order to match the retinal position of the features memorized along a familiar route [40]. Such a simple mechanism based on panoramic images also explains spontaneous biases in ant routes observed in an artificial arena [31].

The present work also supports this hypothesis for route following (see 'Rotational matching of panoramic views' in Results), but suggests that ants may not always follow that strategy. Indeed, in the 32° condition, the ants U-turned and started searching on average half-way to the nest although our analysis of rotational image differences shows that walking in the best matching directions should lead the ants all the way towards the nest or the landmark. We suggest that individual ants may possess a mismatch tolerance threshold that allows them to switch between navigational strategies. If the mismatch is considered too bad, ants might stop such a route strategy (i.e., walking along the best matching direction) and start another strategy. Due to the artificial alteration of the scenery in this situation, this second strategy led the ants to meander in loops, but in a different fashion than the systematic search displayed in a totally unknown area (see Additional file 2). In more naturalistic situations, such a strategy might be adapted for navigation in less familiar environments that do not show excessive mismatch, such as situations in which the ant has been led astray or blown away from her familiar route corridor by a small distance (work in preparation).

Although analysing panoramic images explains a lot, other puzzles remained. We did not manage to explain the early U-turns observed in the 32° left condition. A better knowledge of the nature of insects' perceptions and memories would be precious for further illuminating guidance mechanisms.

### Nature of the insect views?

Do insects use landmarks for guidance? Yes. Much evidence in bees, wasps and ants shows that insects are highly influenced by landmarks (for a review [18]; in *Melophorus bagoti *[9,13]). Do insects focus on individual landmarks only, filtering out the distant panorama from guidance mechanisms? We think not. The present work shows that, even when the dichotomy between proximal landmark and distal panorama is artificially emphasised, guidance is not focused solely on the landmark. But then, are insects' panoramic views constituted of an ensemble of individual landmarks? Probably not. Evidence shows that insects store a pallet of features/parameters like strong boundaries [43], spots of light, centre of gravity, and colour of areas [44] and appear to do so without reconstructing the actual pattern [45]. Insects also have access to landmark distance information based on motion parallax [20-25]. The motion parallax creates a pattern of optic flow that can be used to pinpoint a target location [21]. As with static cues, such dynamic cues can potentially be matched across the whole panoramic view [46] and the insect may not be using them exclusively on isolated landmarks. Rather than isolated landmarks, encoding such a pallet of static and dynamic parameters simultaneously across a large part of the retina seems an appropriate strategy to cope with the complexity of natural scenes and the poor resolution of insects' eyes.

## Conclusion

We have created conditions in which a landmark seemed prominent, easy to extract, and very useful, at least to our primate visual system. But we (primates) have high acuity frontal foveal vision that can be focused on individual objects. Added to that is an entire specialised stream, the so-called ventral stream that is dedicated to object perception [47,48]. To those humans who have seen this landmark, it seemed the obvious one to use. Yet the evidence suggests that the ants did not focus only on the landmark but relied simultaneously on the distant panorama for guidance. Is this pattern peculiar to our experimental situation? We have reasons to think that the use of panoramas as a whole would be more widespread in insect navigation. Using cues that are encoded and processed simultaneously across a large part of the retina can well explain present and past results obtained in ants and seems an appropriate strategy to cope with the complexity of natural scenes, the poor resolution of insects' eyes, and the lack of dedicated object-perception visual streams. It is still unclear what the nature of the parameters is that comprise insects' perceptions and memories, but future studies should not assume that insects functionally segregate landmarks and distal panorama without evidence for such a dichotomy.

## Methods

### Nest area and Landmark

We chose a nest located in an area devoid of any proximal trees and provided the ants with a feeder 10 m away from their nest. The area between the nest and feeder, where the ants navigated, was open, flat, and cleared of any natural debris. The artificial landmark consisted of a huge black sheet (3 m wide and 2 m high) stretched between two poles 90 cm behind the nest entrance (Figure 1). The landmark width subtended an angular size of 118° at the nest location and 15° at the most distant location (i.e., feeder). *Melophorus bagoti *acuity being about 4° [49], the landmark could be perceived all along their homeward route. To the ants, the landmark presented a strong dynamic change in size (increasing in retinal angle of 64° (from 54° to 118°) along the azimuth in the last 2 m of the route). In contrast, the rest of the panorama presented very little apparent displacement, the closest tree being located roughly 14 m away behind the nest. All in all, our artificial landmark stood as an obvious beacon for the nest entrance.

### Protocol

*M. bagoti *lives in the semi-arid terrain of central Australia, which is typically filled with bushes, grass tussocks and trees. The ants were given food *ad libitum* in a fixed feeder 10 m from the nest entrance, and painted at their first visit to the feeder with a colour that marked the day of arrival. After 2 days of spontaneous shuttling between the nest and the feeder, with the artificial landmark immediately behind the nest, the marked foragers were tested. An ant returning from the feeder was captured near the nest and released again at the feeder location with the artificial landmark either left at its original position, removed or displaced by 0°, ± 16°, or ± 32° relative to the feeder-nest direction. Another test consisted of releasing the ants 10 m or 2 m in front of an identical landmark located in a distant test field roughly 100 m away in the same absolute orientation as in the training condition. Ants were tested singly, and each ant was only tested once.

### Path analysis

The training and test fields were covered by a grid of 1-m squares made out of strings stretched between tent pegs that allowed the recording of paths by hand. The recorded paths were digitised into (x, y) coordinates with the software Graphclick™ http://www.arizona-software.ch/ and processed using Matlab™ (Math Works, Natick, MA, USA) programs. Paths were analysed for U-turns and tortuosity. U-turns were defined as walking back at least 20 cm along the Y axis within 50 cm of displacement. The maximum angle away from the training direction that can be travelled for more than 50 cm without being considered as U-turn was thus 113 degrees. The tortuosity index of a path corresponded to the averaged absolute turn angle (in radians) between successive chunks of 20-cm line segments connecting points on the path. A circle of 20 cm radius was placed at the starting point of the digitised path, and where the circle intersected the path defined the first segment. The circle was then placed at the end of segment 1 to define segment 2, etc.

### Panoramic images analysis

To quantify changes in the visual panorama generated by displacing the artificial landmark, 5 reference panoramic pictures (black and white 360*40 pixels) were taken along the trained route, from the feeder to the nest, with the landmark in the training position. For each test condition, with the landmark displaced or removed, we mapped the area explored by the ants with 17 pictures (see Additional file 1). The test pictures were compared to the reference picture that corresponded to the same distance from the feeder. This was always the reference picture that best matched the tested picture. To compare each image to a reference image, we calculated the pixel-wise RMS (route mean square) error for all possible orientations of the reference image. The pixel-wise RMS gives us a value for the mismatch, or image difference, between two images. The RMS of the best matching orientation was recorded for each of the 17 tests pictures and used for the construction of the image difference map (see Figure 2). Interpolation of image differences values using the 17 pictures provided an estimate of mismatch across the whole terrain of travel.

## Competing interests

The authors declare that they have no competing interests.

## Authors' contributions

AW, GB and KC designed the experiment. AW carried out the experiment, performed the analysis and wrote the manuscript. All authors read, revised, and approved the final manuscript.

## Supplementary Material

Additional file 1 **Panoramic picture comparison**. Illustration of panoramic pictures. The recording locations and procedure used to transform and compare them are explained. Click here for file

Additional file 2 **Approaches and searches**. Comparison of the initial approach directions and subsequent search distribution/density in conditions where ants did not reach the landmark. Distinguishes between searches on training and test field. Click here for file

Additional file 3 **Directional switch and first U-turn**. Examples of paths showing segments oriented towards the landmark. Sudden switches in direction and first U-turns are pointed out. Illustrates the independence between direction of travel and 'path uncertainty'. Click here for file
