# Supplementary material for: Landmarks or panoramas: what do navigating ants attend to for guidance?
Source: Front Zool. 2011 Aug 27;8:21. doi: 10.1186/1742-9994-8-21 (PMC3177867; doi:10.1186/1742-9994-8-21)
Supplement: Additional file 1 — Panoramic picture comparison. Illustration of panoramic pictures. The recording locations and procedure used to transform and compare them are explained. [file 1742-9994-8-21-S1.PDF]

Landmarks or panoramas: what do navigating ants attend to for guidance?

Additional file 1

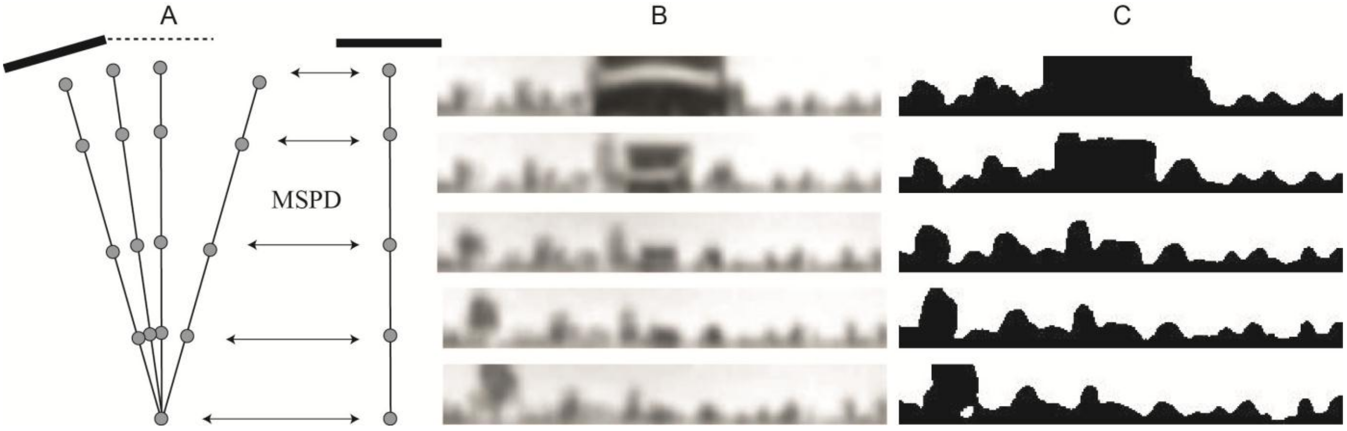

Panoramic picture comparisons. A. The grey circles indicate the locations where the 360° pictures were recorded in training and test conditions. Each picture from the test condition was compared (MSPD = mean squared pixel difference (see [1] for more details)) with the most similar training picture. The picture resolution was set to 4 pixel/degree (B) in order to approach the insects' visual acuity, and then transformed into black and white (C) to avoid bias due to variations in overall light levels. Such a transformation picks out the skyline (top elevations of terrestrial objects) as key information, consistent with recent findings on this species [2].

1. Zeil J, Hofmann MI, Chahl JS: **Catchment areas of panoramic snapshots in outdoor scenes.** *J Opt Soc Am* 2003, **20:**450-469.
2. Graham P, Cheng K: **Ants use the panoramic skyline as a visual cue during navigation.** *Curr Biol* 2009, **19:**R935-R937.
