# Supplementary material for: Landmarks or panoramas: what do navigating ants attend to for guidance?
Source: Front Zool. 2011 Aug 27;8:21. doi: 10.1186/1742-9994-8-21 (PMC3177867; doi:10.1186/1742-9994-8-21)
Supplement: Additional file 2 — Approaches and searches. Comparison of the initial approach directions and subsequent search distribution/density in conditions where ants did not reach the landmark. Distinguishes between searches on training and test field. [file 1742-9994-8-21-S2.PDF]

Landmarks or panoramas: what do navigating ants attend to for guidance?

Additional File 2

Large displacements and removal of the landmark: approaches and searches.

With large displacements (Rotation 32 Right and 32 Left) or removal of the landmark (No landmark), ants ran a rather straight initial segment and then displayed a U-turn on average half way from the nest and started searching. We considered here the first U-turn displayed as marking the end of the initial approach and the beginning of the search. Initial approaches tended to be a little bit skewed towards the displaced landmark (t-test against midline significant only for Rotation 32 Right at 5 m: $t=-4.2479$, $p<0.001$,) (A). The subsequent searches appeared on average centred on the first U-turn in all three groups (paired t-test: search centre of gravity vs. U-turn position: along x_axis: p's>0.253 (B); along y-axis: p's>0.431). Interestingly, the search scatter varied significantly across condition (ANOVA $F=8.7$, $p<0.001$). A displacement of the landmark to the left (Rotation 32 Left) led to slightly more scattered searches than the removal of the landmark (No landmark). Strikingly, searches displayed on the modified training field (Rotation 32 Right, 32 Left and No landmark) were much more scattered than searches displayed on the unfamiliar test field (Distant field) (C). This shows that ants' search behaviour is not a rigid strategy but is adjusted according to the view perceived. The view perceived on the modified training field must provide a better match to the route memorised during training than the view perceived on the distant test field does. Such a better match might induce the ants to carry on further away before turning, leading to a more widely spread search. Within the training field conditions, displacement of the landmark results in a slightly flatter gradient of image differences on the landmark side. This may explain why searches with the displaced landmark were slightly more spread than when the landmark was removed. In contrast, on the distant field, strong mismatches may induce the ants to turn and thus display tighter loops. Interestingly, the search patterns displayed on the distant field appeared slightly skewed towards the bottom left (see Figure 4.B), suggesting that such characteristic systematic searches displayed in unfamiliar environment [1] might also be in part driven by vision.

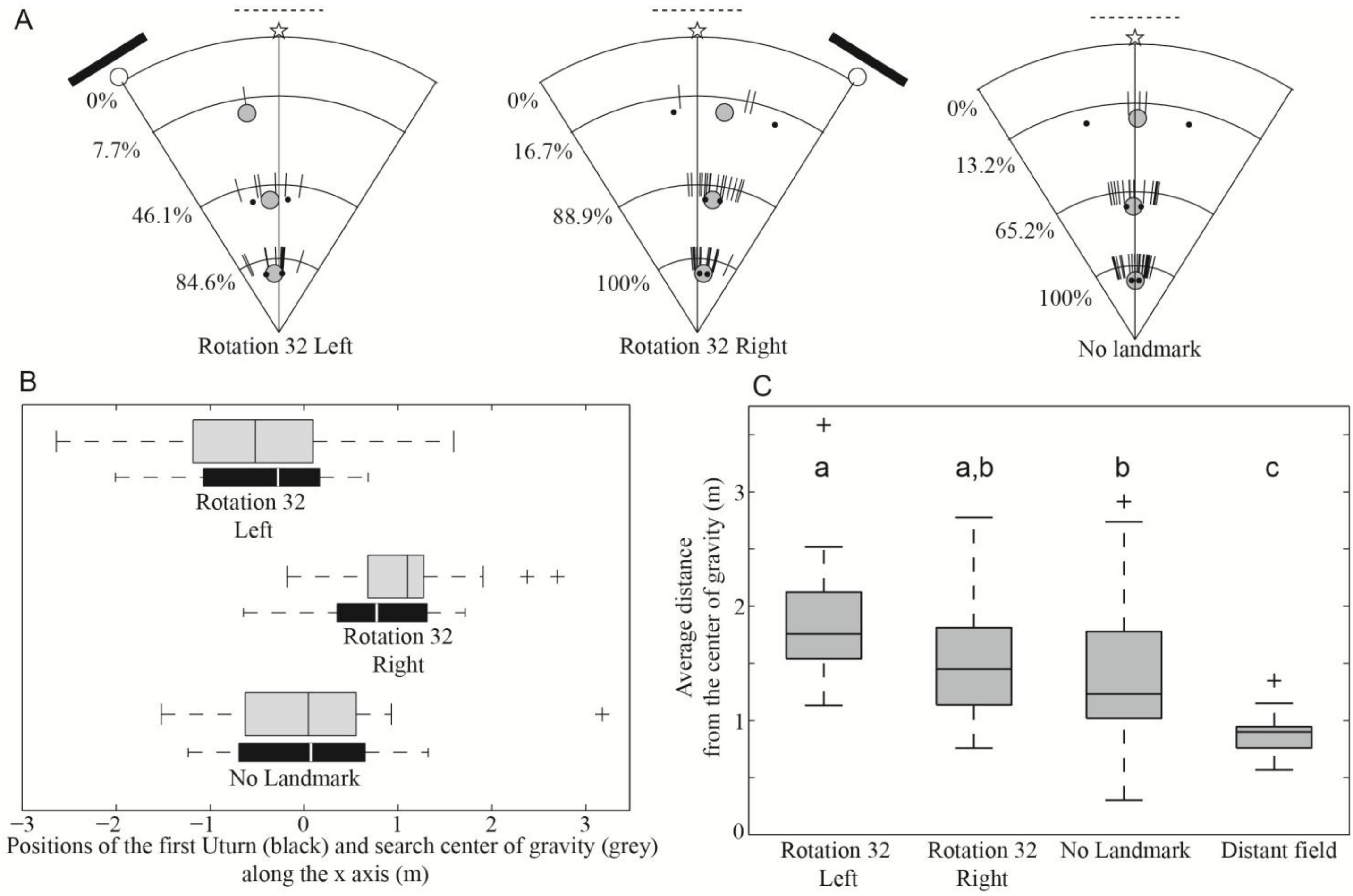

A. Lateral deviation of the individual ants at 2 m, 5 m, 8 m, and 10 m away from the feeder during their initial approach (i.e., before the first U-turn). The big grey circles indicate the average direction and the small black dots indicate the 95% confidence interval. The numbers on the side indicate the percentage of ants that reached that distance before displaying a U-turn. Bar: beacon position during test. Dashed bar: beacon position during training. Stars: nest position. Open circle: fictive nest position relative to the beacon. B. Distribution of the lateral positions of the ants' first U-turn (black) and subsequent search centre of gravity (grey). C. Distribution of the individuals' search spread. The spread was measured as the average distance of the search from its centre of gravity. Test groups with identical letters are not significantly different by Tukey's post hoc test. B,C) Whiskers of the boxplot extend to the most extreme data points not considered outliers, and outliers (i.e., individuals further than 1.5 × interquartile-range away from the closest quartile) are plotted individually (crosses).

1. Schultheiss P, Cheng K: **Finding the nest: inbound searching behaviour in the Australian desert ant, *Melophorus bagoti*.** *Anim Behav* 2011, **81:**1031-1038.
