# Supplementary material for: Landmarks or panoramas: what do navigating ants attend to for guidance?
Source: Front Zool. 2011 Aug 27;8:21. doi: 10.1186/1742-9994-8-21 (PMC3177867; doi:10.1186/1742-9994-8-21)
Supplement: Additional file 3 — Directional switch and first U-turn. Examples of paths showing segments oriented towards the landmark. Sudden switches in direction and first U-turns are pointed out. Illustrates the independence between direction of travel and 'path uncertainty'. [file 1742-9994-8-21-S3.PDF]

Landmarks or panoramas: what do navigating ants attend to for guidance?

Additional file 3

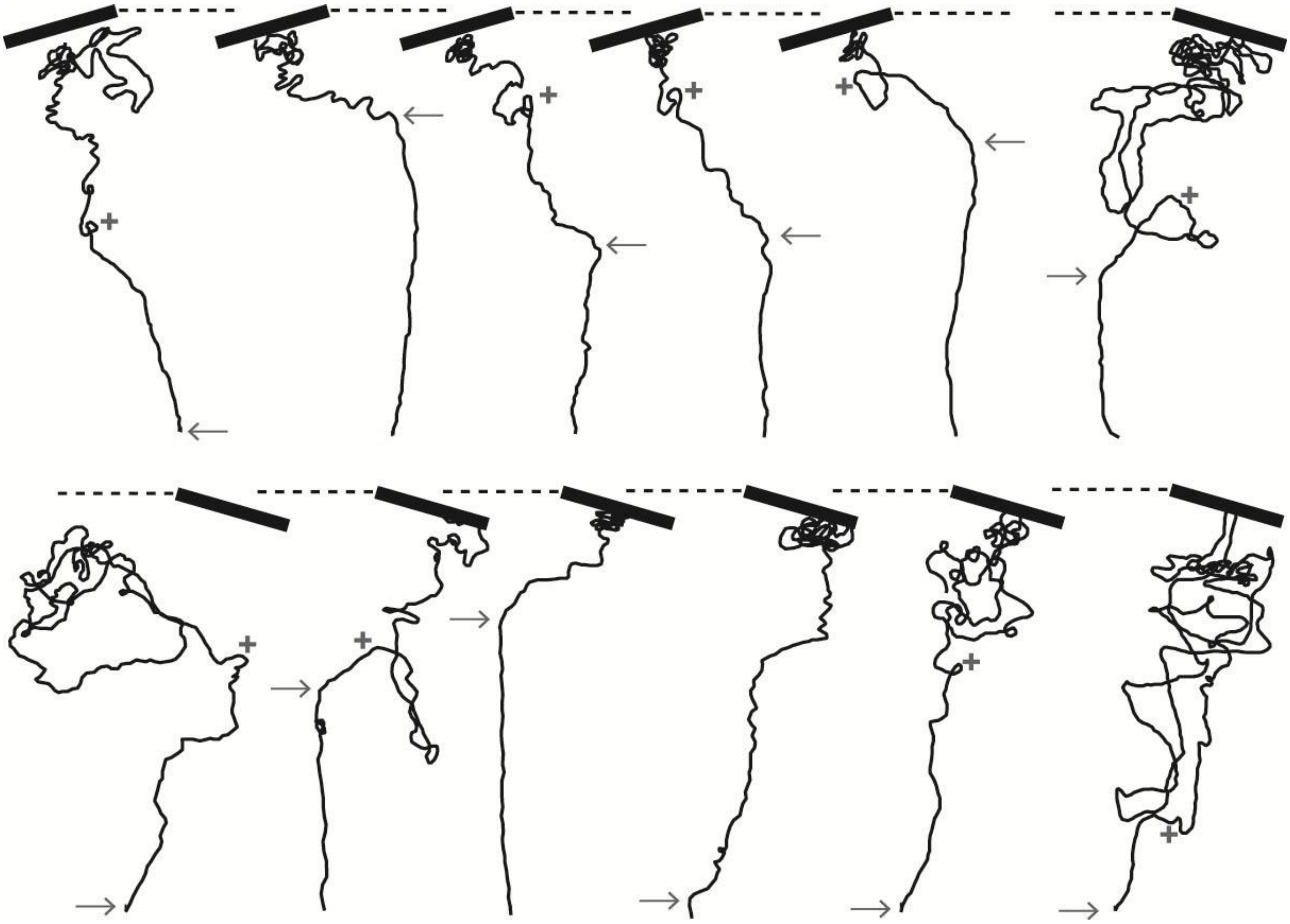

Directional switch and first U-turn. Paths from individuals that headed towards the 16° displaced landmark (black bar) from the beginning or after a neat transition in the direction of travel. Grey arrows indicate the beginning of the segment oriented towards the landmark and crosses indicate the presence of a first U-turn along the approach to the landmark. Behaviours indicative of 'uncertainty' such as U-turn or high meandering do not occur immediately after the switch towards the landmark as it would be expected if the path disruption was due to the new compass direction of travel. Previous work showed that *M. bagoti* ants can readily match and use familiar panorama presented in a wrong compass direction [1]. Dashed line indicates the position of the landmark during training.

1. Graham P, Cheng K: **Ants use the panoramic skyline as a visual cue during navigation.** *Curr Biol* 2009, **19:**R935-R937.
